# A Case of Neonatal Chlamydial Conjunctivitis: Illustrating the Typical Aspects of Presentation and the Importance of Empiric Treatment and a Multidisciplinary Approach

**DOI:** 10.7759/cureus.64463

**Published:** 2024-07-13

**Authors:** Imani Nwokeji, Kun Ding, Scott Ketner

**Affiliations:** 1 Ophthalmology, City University of New York (CUNY) School of Medicine, New York, USA; 2 Ophthalmology, BronxCare Health System, New York, USA

**Keywords:** neonatal conjunctivitis, pediatrics, treatment, prophylaxis, infectious

## Abstract

Chlamydia trachomatis is the most common cause of infectious neonatal conjunctivitis in the United States and worldwide. If left untreated, it can cause scarring of the cornea or conjunctiva. Furthermore, neonatal chlamydial conjunctivitis is not infrequently associated with chlamydial pneumonia, making this type of neonatal conjunctivitis important to recognize and treat. We present a case of neonatal chlamydial conjunctivitis that occurred despite routine prenatal screening and the use of erythromycin ophthalmic ointment at birth. The case illustrates many of the typical aspects of the presentation of this condition as well as the importance of empiric treatment and a multidisciplinary approach, involving not only ophthalmology and pediatrics but infectious diseases and social services, when appropriate.

## Introduction

Chlamydia trachomatis is the most common cause of infectious neonatal conjunctivitis in the United States and worldwide [1,2]. If left untreated, it can cause scarring of the cornea or conjunctiva [3,4]. Furthermore, neonatal chlamydial conjunctivitis is not infrequently associated with chlamydial pneumonia [5,6], making this type of neonatal conjunctivitis important to recognize and treat. In the United States, where routine prenatal screening and treatment are the standard of care, neonatal chlamydial conjunctivitis is rare, occurring in an estimated 8.5 cases per 100,000 live births [7]. We present a case of neonatal chlamydial conjunctivitis that occurred despite routine prenatal screening and the use of erythromycin ophthalmic ointment at birth. The case illustrates many of the typical aspects of the presentation of this condition as well as the importance of empiric treatment and a multidisciplinary approach, involving not only ophthalmology and pediatrics but infectious diseases and social services, when appropriate.

## Case presentation

**Figure 1:**
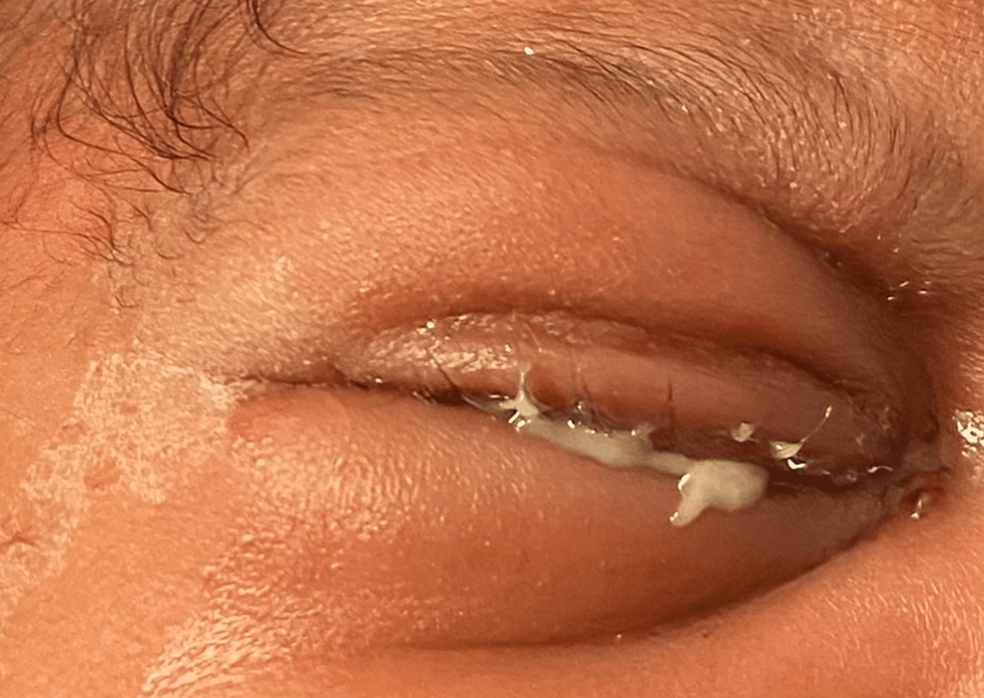
Infant at presentation with right upper and lower eyelid edema, erythema, and discharge

**Figure 2:**
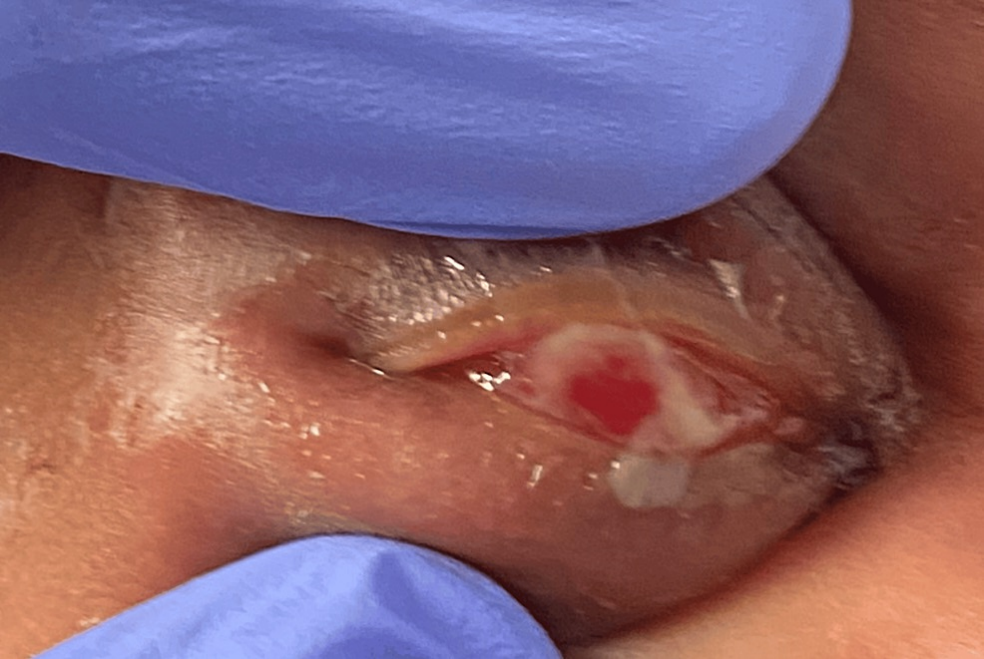
The conjunctiva is injected, discharge is present, and the upper eyelid is unintentionally everted. The cornea was intact without any staining on fluorescein testing

A 16-day-old male infant was brought to the emergency department (ED) by his parents due to right eye swelling and redness for about five days, as well as bloody tears since earlier in the day. The baby’s mother had undergone routine prenatal screening, including negative chlamydial and gonococcal results, and the baby had undergone routine use of erythromycin ophthalmic ointment at birth. Examination in the ED showed erythematous and edematous right upper and lower eyelids with mucopurulent discharge on the lashes and diffuse conjunctival injection (Figures 1-2). There was no corneal staining. There was no obvious involvement of the fellow eye (left eye). A dilated fundus examination of both eyes revealed no abnormalities. Gram stain as well as bacterial, chlamydial, and viral eye cultures were sent. Ophthalmology recommended empiric, systemic treatment for possible *Neisseria gonorrhoeae* or chlamydia, erythromycin ophthalmic ointment, saline lavage, infectious disease consultation, and admission. Per pediatrics, there were no signs of systemic disease.

The baby was treated empirically with a one-time dose of intramuscular injection of ceftriaxone (50 mg/kg) in the ED to cover for possible gonococcal conjunctivitis. The following day, the primary team started a three-day course of oral azithromycin (20 mg/kg daily) per consultation with infectious diseases, who echoed ophthalmology’s recommendation for empiric treatment, i.e., to cover for possible chlamydia. Pharyngeal swabs for chlamydia and gonorrhea polymerase chain reaction (PCR) testing were sent by pediatrics within 24 hours of admission.

The baby’s condition appeared possibly somewhat improved when the baby was assessed by ophthalmology approximately 24 hours after the initial presentation, and the examination was significantly improved when the baby was examined by ophthalmology approximately 72 hours after the initial presentation. The baby received a total of three days of systemic azithromycin treatment per infectious diseases’ recommendations; no pneumonia or symptoms of systemic involvement developed. Results from the baby’s gram stain returned approximately 12 hours after the initial presentation and showed leukocytes and gram-negative bacilli. Subsequent results from bacterial eye culture showed gram-positive bacilli. Ultimately, the bacteria growing on the bacterial eye cultures were identified as *Fusobacterium mortiferum* and *Streptococcus viridans* (the former growing on the anaerobic culture and the latter growing on the aerobic culture). The chlamydia results from the PCR test performed on the pharyngeal swab were positive, but they were not received until days after the baby’s presentation, by which time the baby’s eye examination had significantly improved and the condition was clearly resolving. Because the baby’s eye examination had shown steady improvement after the empiric treatment with systemic azithromycin and ceftriaxone, the bacteria identified on the gram stain and bacterial eye cultures (i.e., *Fusobacterium mortiferum* and *Streptococcus viridans*) were not ultimately thought to be responsible for the infection; however, to be cautious, tobramycin ophthalmic ointment was added at the time to the baby’s regimen of erythromycin ophthalmic ointment for some days. A social services consultation was placed due to a number of factors: the baby’s mother’s age (16 years old), the baby’s parents’ failure to bring the baby into the ED for five days after the symptoms started, and the baby’s parents’ largely being absent from the hospital while the baby was admitted.

Ultimately, with the involvement of the city’s Administration for Children’s Services, custody of the baby was removed from the baby’s parents and given to the baby’s maternal grandmother. Given the baby’s positive chlamydia testing, it was recommended that counseling be provided to the baby’s parents regarding the chlamydia test results and obtaining treatment, as well as testing for other sexually transmitted diseases. The baby was discharged approximately one week after the initial presentation, and the infection appeared essentially resolved at ophthalmology outpatient follow-up approximately one week after discharge, i.e., approximately two weeks after the initial presentation.

## Discussion

Cases of infectious neonatal conjunctivitis can cause vision loss if not treated promptly and can be associated with systemic infection with the pathogen. For these reasons, recognition and prompt, empiric treatment are important. In this case, the baby’s development of symptoms at approximately 11 days of age (i.e., approximately five days prior to presentation, per history), the baby’s mucopurulent discharge, and bloody tears were all consistent with chlamydial neonatal conjunctivitis. Neonatal conjunctivitis that occurs within the first 24 hours of birth is most likely chemical, whereas conjunctivitis occurring within 24-48 hours of birth is more likely gonococcal, and conjunctivitis occurring within 5-14 days is more likely chlamydial [1,2,8]. Herpes simplex neonatal conjunctivitis typically presents one to two weeks after birth [9] and can be associated with generalized herpes infection; vesicles around the eye and corneal involvement are common [10]. However, all of these conditions can, of course, present in atypical ways, and gonococcal conjunctivitis in particular can cause rapid damage, including corneal ulcers and perforation, if not treated promptly. Given that the gram stain and cultures can take some time to return, empiric treatment is vital. Also vital is the involvement of an infectious diseases service for the evaluation and management of any systemic involvement, as well as the evaluation and treatment of the baby’s parents, when an infectious cause is identified. Social services consultation is sometimes warranted, as in this case.

## Conclusions

Appropriate management of neonatal conjunctivitis involves prompt evaluation, empiric treatment when warranted, and a multidisciplinary approach.

## References

[1] Makker K, Nassar GN, Kaufman EJ (2024). Neonatal conjunctivitis. StatPearls [Internet].

[2] Zikic A, Schünemann H, Wi T, Lincetto O, Broutet N, Santesso N (2018). Treatment of neonatal chlamydial conjunctivitis: a systematic review and meta-analysis. J Pediatric Infect Dis Soc.

[3] Goscienski PJ, Sexton RR (1972). Follow-up studies in neonatal inclusion conjunctivitis. Am J Dis Child.

[4] Forster RK, Dawson CR, Schachter J (1970). Late follow-up of patients with neonatal inclusion conjunctivitis. Am J Ophthalmol.

[5] Hammerschlag MR, Chandler JW, Alexander ER, English M, Koutsky L (1982). Longitudinal studies on chlamydial infections in the first year of life. Pediatr Infect Dis.

[6] Tipple MA, Beem MO, Saxon EM (1979). Clinical characteristics of the afebrile pneumonia associated with Chlamydia trachomatis infection in infants less than 6 months of age. Pediatrics.

[7] (2002). Sexually transmitted diseases treatment guidelines 2002. Centers for Disease Control and Prevention. MMWR Recomm Rep.

[8] Singh G, Galvis A, Das S (2018). Case 1: eye discharge in a 10-day-old neonate born by cesarean delivery. Pediatr Rev.

[9] Yu K, Epley KD, Bowman KM, Prakalapakorn G, Prabhu S, Griffiths D, Nguyen A (2024). Neonatal conjunctivitis. https://eyewiki.org/Neonatal_Conjunctivitis.

[10] Mallika P, Asok T, Faisal H, Aziz S, Tan A, Intan G (2008). Neonatal conjunctivitis - a review. Malays Fam Physician.

